# The Law of Illinois Concerning Insane Persons

**Published:** 1878-11

**Authors:** R. J. Patterson

**Affiliations:** Batavia, Ill.


### Domestic Correspondence.

### THE LAW OF ILLINOIS CONCERNING INSANE PERSONS.

The following open letter has been addressed to the Board of State Commissioners of Public Charities of the State of Illinois, Hon. George S. Robinson, President, by Dr. R. J. Patterson, of Batavia, Illinois:

The law of Illinois regulating the commitment of insane persons to hospitals is peculiar. The like of it does not exist in any other State or country.

Your Board of Public Charities, to which, in a special degree, the welfare of the insane is by law committed, is properly looked to for any suggestions in regard to needed changes in this law; and in the absence of any suggestions, changes are not likely to be made.

The law appears to me to be objectionable from a medical point of view, and I deem it proper, therefore, to call your attention to some of its objectionable features:

*The Trial by Jury.* It is not claimed by anyone that the jury trial confers any protection or other benefit upon the really insane. It is humiliating, offensive and hateful to those of them who have not lost nearly all perception and sensibility. It is also odious to the family and friends of the insane, as shown by the fact that they often seek to evade it.

It is alleged that the jury trial is necessary to protect sane persons from imprisonment by mistake or fraud.

I reply, that the jury trial has not been found necessary or desirable in other States or countries; and the evils sought to be avoided, if they ever existed, must be of exceedingly rare occurrence, and that mistakes and fraud are possible, and do actually exist, under the present law.

The following letters show that persons have been tried by jury and committed to hospitals who were not insane. They also show that persons who were really insane have been subjected to the ordeal of more than one trial, because of the incompetency of the jury to weigh the evidence which proved the insanity; therefore, neither the personal liberty of the sane nor the welfare of the insane is secured by the present law. So easy has it been found to either deceive or suborn a jury, that simulating criminals gain admission to hospitals as means of escape, while the insane are kept waiting:

ILLINOIS CENTRAL HOSPITAL FOR THE INSANE,
SUPERINTENDENT'S OFFICE, JACKSONVILLE, Ills., Sept. 11, 1878.

DR. R. J. PATTERSON—*Dear Sir:* Your letter of the 9th inst. is received In answer to your first question, as to whether any person or persons, who were not insane, have been declared insane by a jury, I would say, I have in mind two cases. Both were arrested for some criminal act, showed symptoms of insanity while in jail, were "tried for insanity," and, by a jury, found insane. Both were sent here, and both escaped, after a brief stay with us. I think neither was insane.

Answer to second question: It has not unfrequently come to my knowl edge, where persons have been subjected to a second trial, before the neces sary verdict to commit them to the hospital could be obtained.

Yours, very truly, H. F. CARRIEL.

NORTHERN HOSPITAL FOR THE INSANE,
ELGIN, Ill., Sept. 17, 1878

DR. R. J. PATTERSON, BATAVIA, Ill.—*My Dear Doctor:* Replying to your letter of inquiry of the 9th inst., I have to state that during the past five years four men and two women, resident in adjoining counties, have been adjudged insane upon jury trial before our county courts, in accordance with the requirements of the statutes of this State, and duly committed to this hospital as "fit persons for detention and treatment here," who, upon investigation, were found to be "*not insane*," and consequently dismissed from the custody of the institution and given their full liberty again. I will state further, that many instances have come to my knowledge where *insane persons* have been subjected to *two or three jury trials* before being declared insane, and this in the face of the clearest evidence as to their derangement, if analyzed by those competent to weigh testimony of this kind. Of course this is all *wrong*, as it not only retards treatment in the early stages of the disease—its most hopeful period—but wounds, nay, outrages the finest sensibilities of our being, and works an injury to the insane and their friends that is well nigh irreparable. I should be glad to see some change in the law that would do away with these "criminal proceedings." Very truly, yours,

EDWIN A. KILBOURNE.

The above letters, from the medical superintendents of two of our State hospitals, are high testimouy. It is beyond a reasonable doubt that no less than eight persons have been committed to two of our State hospitals by jury trials who were not insane. I challenge anyone to show such monstrous abuses under any other legal proceedings in the history of the State.

It is a fact well known to the medical profession, that very early treatment is the most important factor in the restoration of insane persons to health. It is generally admitted, also, that the well conducted modern hospital affords the best known means for successful treatment. Whatever, therefore, puts a hindrance in the way of the promptest admission to early treatment of the insane vitally injures them.

The jury trial in open court, having the forms of a criminal procedure, the publicity given to it, the crowd of spectators often present, the delicate nature of the testimony sometimes demanded, the public exposure of infirmities which are the offspring of disease and not of crime, the danger in some cases to health and life from exposures, all tend to render the jury trial so objectionable and so offensive to the family and friends of the insane, that commitment to a hospital is likely to be delayed until the last degree of endurance has been reached, and the most favorable period for successful treatment has passed. Our large hospitals and our poor-houses are over-crowded with the chronic incurable insane, notwithstanding the fact that the State has provided for the care of more than 1,000 patients within the last ten years, while the present law has been in force. Is it not possible that the detestation in which the law is held, and the consequent hindrance to early treatment, may afford, in part at least, an explanation of the large number of incurable cases at last thrown upon the State and county for support?

The law, instead of facilitating the attainment of the promptest protection and relief to those who are helpless and dependent because of dethronement of reason, makes those who are exempt from mental maladies and disabilities, the special objects of its paternal solicitude—though not one single authenticated case of abuse under previous laws had been adduced as a reason for departing from such legal proceedings as were in accordance with science and humanity.

If anyone asks why the law is shocking to the sensibilities of the insane and their friends, let him attend some of these trials, or glance at the reports of them in the daily newspapers. In the Chicago *Tribune* of June 21, 1878, appeared the following paragraph:

"In the County court, yesterday morning, a number of insane cases were called. The industry of the "*professionals*" in trying to get on the jury to try these unfortunates, on account of the two dollars in the job, was amusing, and was commented on quite freely. Some of the "professionals" succeeded in getting places, and, after considerable delay, the trials proceeded."

Another paragraph in the Chicago *Tribune* of August 22d read as follows:

"To-day is insane day in the County court, but, the judge being absent, no trials will be had. The insane now in jail, and around the police stations, must wait. Somebody is to blame, and cannot be blamed too severely. The unfortunates have committed no crime that they should remain in prison. A year ago, during the adjournment of the County court, all such cases were disposed of in the Criminal court."

Thus Illinois, in her zealous care to protect sane people, imprisons in a felon's cell insane persons for an indefinite period, awaiting the execution, possibly in a criminal court, of the provisions of her sardonic law.

On the 11th of June, 1878, eleven cases were set for trial. The "professionals" doubtless succeeded in gettting on the jury and obtaining the two dollars in the "job." Of these eleven defendants, he whose case was called last, was possibly compelled to listen not only to the evidence in his own case, but to the incoherent vociferations as well as the testimony in regard to the ten other defendants whose trial proceded his. Comment is unnecessary, further than to say that such treatment and exposure of the insane is not humane or even decent, and it seems incredible that any right-minded man of ordinary perceptions and sensibilities should regard such court-room scenes with feelings other than those of detestation.

The laws of England and Scotland in regard to the commitment of insane persons to hospitals, are the result of long experience and intelligent observation. In England, insane persons are committed to hospitals upon the certificate of two physicians, and the written order or request of nearest relatives, friends or guardians. In Scotland, in addition to the forms required in England, the intervention and order of a magistrate is required. Nowhere in Christendom is the trial by jury thought necessary, except in the State of Illinois.

The Earl of Shaftsbury, for many years chairman of the English Lunacy Commission, recently gave evidence before a parlimentary committee of inquiry as to the lunacy laws and condition of the insane, as follows:

"What I state shows the absolute necessity of paying great attention to the earliest stage of the disorder; and though I could by no means render admission into the asylums more easy than it is, I most undoubtedly would not render it more difficult."

When asked directly as to his views of the Scotch system of the interposition of a magistrate (page 518) and its incorporation with English law, he says: "I am sure it would be most repugnant to our tastes and feelings to have the civil magistrate interpose in these matters." "It would be no protection whatever." "I cannot conceive of anything which to my mind would be worse. I will do anything I can in the world to protect the patient; but I know if I were to assent to do what is proposed, I should assent to that which would be doing him an irreparable evil."

C. S. Percival, secretary of the Commissioners of Lunacy, says: "In my opinion the medical certificates are the most important safeguards to the personal liberty of the subject, and the present forms are sufficient."—Page 461.

Dr. Fox says: "I do not think the intervention of a public officer would be of any material value at all to the liberty of the subject; it would oppose an additional difficulty to the earlier treatment of insanity, which is so very important."—Page 463.

Mr. Wilkes, Commissioner in Lunacy, says: "The present law is quite sufficient to protect the personal liberty of the people."—Page 265.

Dr. Lockhart Robertson would have as a commissioner "a leading physician in the district (asylum district) to renew the medical certificates upon which the patient was originally admitted."—Page 467.

Dr. Bucknill says: "I think the principle should be to make the admission as easily as possible, to provide for early treatment."—Page 473.

Dr. Maudsley "was strongly of the opinion that the present forms for the admission of private patients are quite sufficient, and, if made more stringent, would operate injuriously to their early treatment and chances of recovery."—Page 490.

"If it is considered desirable, as I have heard suggested, that the medical certificates go before some public officer before they are acted upon, it seems to me no public officials would be better qualified than the commissioners (of lunacy), to whom exact copies are now sent within twenty-four hours. If the matter were really entered into in each case, it would be a very anxious responsibility—a formidable matter to undertake; if not, it would simply become a mere matter of routine, which, adding to the publicity, and adding to the expense, and adding to the delay of getting a patient under care, would make early treatment more difficult than it is."—Page 494.

Question 3744: "You think that if there was more care taken, or more delay in admitting or consigning patients to asylums, their cure would be more doubtful?"

Answer: "Undoubtedly, there are two great objects to be kept in view with regard to the detention of patients; they are put under care, not only for their own safe custody, because they are dangerous to themselves or others, but another and most important object, if insanity is to be cured, is, that they be put under care for treatment, and early, because recoveries are entirely in proportion to the early stage in which treatment is adopted. If regulations are made more stringent than they are now—and indeed the present regulations operate, to some extent, in that direction—the friends of patients will, instead of sending them from home, as is almost essential in a case of insanity—unlike, in this respect, other diseases—keep them at home under improper conditions, and so very much injure their chance of recovery."—Page 492.

"It is my experience, as a physician, that the friends shrink very much from that [going through forms]. They dislike the supposed publicity of it; they dislike the formality pronouncing him a lunatic; and they will not remove him in consequence."—Page 492.

Dr. Mortimer Granville says: "I think the best plan would be for a patient to be sent, under ordinary circumstances, to an ordinary hospital, immediate notice being given to the Commissioners in Lunacy, who would instruct some official on their behalf to visit and certify to them the condition of the patient and expediency of retaining him."—Page 502.

Mr. Charles P. Phillips, Commissioner in Lunacy, said: "In twelve years of experience as a Commissioner in Lunacy, he had not known a case where a person, being sane, had been improperly confined in an asylum."

Those of the medical profession in the United States who have given most thought to the welfare of the insane, and who are, therefore, most entitled to hold opinions, substantially agree with the Earl of Shaftsbury, the English Lunacy Commision, and the medical profession in England and Scotland.

Every physician knows that the removal of certain cases of puerperal insanity to court-houses in cold weather, to localities distant from the patient's house, may endanger not only the recovery but the life of such patient. There are risks, it is true, from exposure in removing them to a hospital—but this is a necessity. No humane reason can be assigned for an added risk which in some cases is little less than homicidal.

The law is objectionable, because the jury trial exposes to public comment in detail, family diseases, infirmities and infelicities which the public have no claim to know.

The law is objectionable, in that it debars non-residents the privilege of receiving treatment at *private expense* within the boundaries of the State. If an insane person should be brought by friends for temporary treatment at private expense, from the State of Wisconsin to any place in Illinois where insane persons of the private class are treated, he cannot receive treatment without the jury trial and this cannot be obtained, the courts having no jurisdiction in such cases. This proscription bears heavily on the patient. It is not humane, nor does it regard the comity which should exist between States.

In short, the law, by its hard condition, practically works a hindrance to prompt treatment of the insane while there is most hope of recovery, and thus tends to fill our hospitals with incurable cases. It is my belief that it entails upon the State a life-long support of many, and a life-long suffering upon some who might be restored to health by the earliest and best treatment.

Allow me respectfully to suggest to the Board of State Charities, which has the welfare of the insane to a special degree in its keeping, that the personal liberty of the individual may be preserved more fully than it now is, and yet the evils of the jury trial in cases of insanity done away with. I would by no means jeopard the liberty of the individual, but would make it more safe than it now is, by leaving the question of insanity with those equally honest and more competent than the average juryman.

In the State of New York, the requirements of the law, which are especially commendable, for the commitment of an insane patient to an asylum are:

1. A certificate of two physicians, under oath, setting forth the insanity of such person.

2. That the physicians signing the certificates must be duly qualified as medical examiners in lunacy, by being certified by a judge of a court of record as possessing the requisite qualifications; that the certificates shall be made on personal examinations of the patient, in accordance with forms prescribed by the State Commissioner in Lunacy, and bear date of not more than ten days prior to the commitment.

3. The certificates must be approved by the Judge of the county or district in which the patient resides.

On account of those who see safety only in the jury trial, this may be *permitted* in every case, but it should not be *compulsory* in every case.

I will suggest a return, in part only, to proceedings under the old law. Unless the New York law is satisfactory, I would require, as a prerequisite of admission to a hospital, in each case, the sworn certificate of a commission, to be appointed by any court of record, this commission to be composed of two physicians and a lawyer. It should be the duty of the commission to visit the person alleged to be insane, and to report, in writing, directly to the court, its opinions and the reasons for them. The court, in its discretion, may approve the finding of the commission. In case the court should fail to be satisfied with the report of the commission, it should have power to call a jury, and should be required to do so whenever the trial is demanded by the person alleged to be insane, or by any citizen of the State.

A law after this project would not be less exacting than the most rigid code of any of the United States, nor that of England or Scotland. Respectfully,

R. J. PATTERSON, M. D.

BATAVIA, ILL., October 1, 1878.

---

THE librarian of the Chicago Medical Press Association hereby acknowledges the receipt, from the Woman's Hospital Medical College of Chicago, of a collection of books, consisting of 44 bound volumes and 158 Journals.

